# Cross-Talk Between Iron and Glucose Metabolism in the Establishment of Disease Tolerance

**DOI:** 10.3389/fimmu.2018.02498

**Published:** 2018-10-30

**Authors:** Ana Rita Carlos, Sebastian Weis, Miguel P. Soares

**Affiliations:** ^1^Instituto Gulbenkian de Ciência, Oeiras, Portugal; ^2^Department of Anesthesiology and Intensive Care Medicine, Jena University Hospital, Jena, Germany; ^3^Institute for Infectious Disease and Infection Control, Jena University Hospital, Jena, Germany; ^4^Center for Sepsis Control and Care, Jena University Hospital, Jena, Germany

**Keywords:** iron metabolism, glucose metabolism, anorexia of infection, disease tolerance, nutritional immunity

## Abstract

Infectious diseases are associated with disruption of host homeostasis. This can be triggered directly by pathogens or indirectly by host immune-driven resistance mechanisms. Disease tolerance is a defense strategy against infection that sustains host homeostasis, without exerting a direct negative impact on pathogens. The mechanisms governing disease tolerance encompass host metabolic responses that maintain vital homeostatic parameters within a range compatible with survival. Central to this defense strategy is the host's ability to sense and adapt to variations in nutrients, such as iron and glucose. Here we address how host responses regulating iron and glucose metabolism interact to establish disease tolerance and possibly modulate resistance to infection.

## Introduction

**Figure 1:**
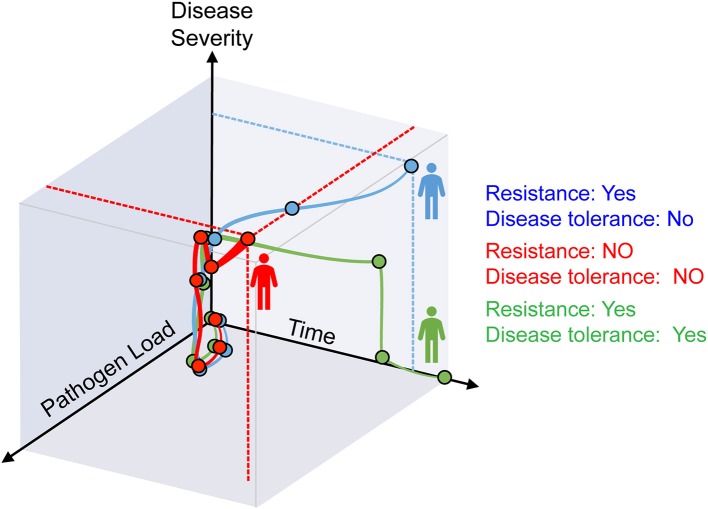
Resistance and disease tolerance to infection. As host pathogen load increases during infection, disease symptoms become apparent and give rise to the clinical signs of infectious diseases. After an initial phase where both pathogen load and disease severity increase, the three possible outcomes are: (i) host homeostasis prevails based on resistance and disease tolerance mechanisms that eliminate pathogens and sustain vital metabolic outputs (green), (ii) resistance mechanisms reduce pathogen load but tissue damage control mechanisms fail to establish disease tolerance, compromising host homeostasis (blue); (iii) resistance mechanisms fail to control pathogen burden and tissue damage control mechanisms fail to establish diseases tolerance, compromising host homeostasis (red).

Avoidance, resistance, and disease tolerance are evolutionarily conserved defense strategies that limit the negative impact of pathogens on host health and fitness (1). Avoidance limits exposure to exogenous pathogens and resistance expels, neutralizes or destroys invading pathogens, while disease tolerance acts without interfering directly with pathogens (1, 2) (Figure 1).

Disease tolerance relies on stress and damage responses that confer tissue damage control (3), that is, support the functional output of host tissues as a means to maintain vital homeostatic parameters within a range compatible with survival to infection (2, 4, 5). Stress and damage responses sense and react to variations in environmental cues or to damage imposed to cellular macromolecules and organelles, respectively (3). These are essential to provide metabolic adaptation to the stress and damage imposed directly by pathogens or indirectly by immune driven resistance mechanisms (3, 4).

**Figure 2:**
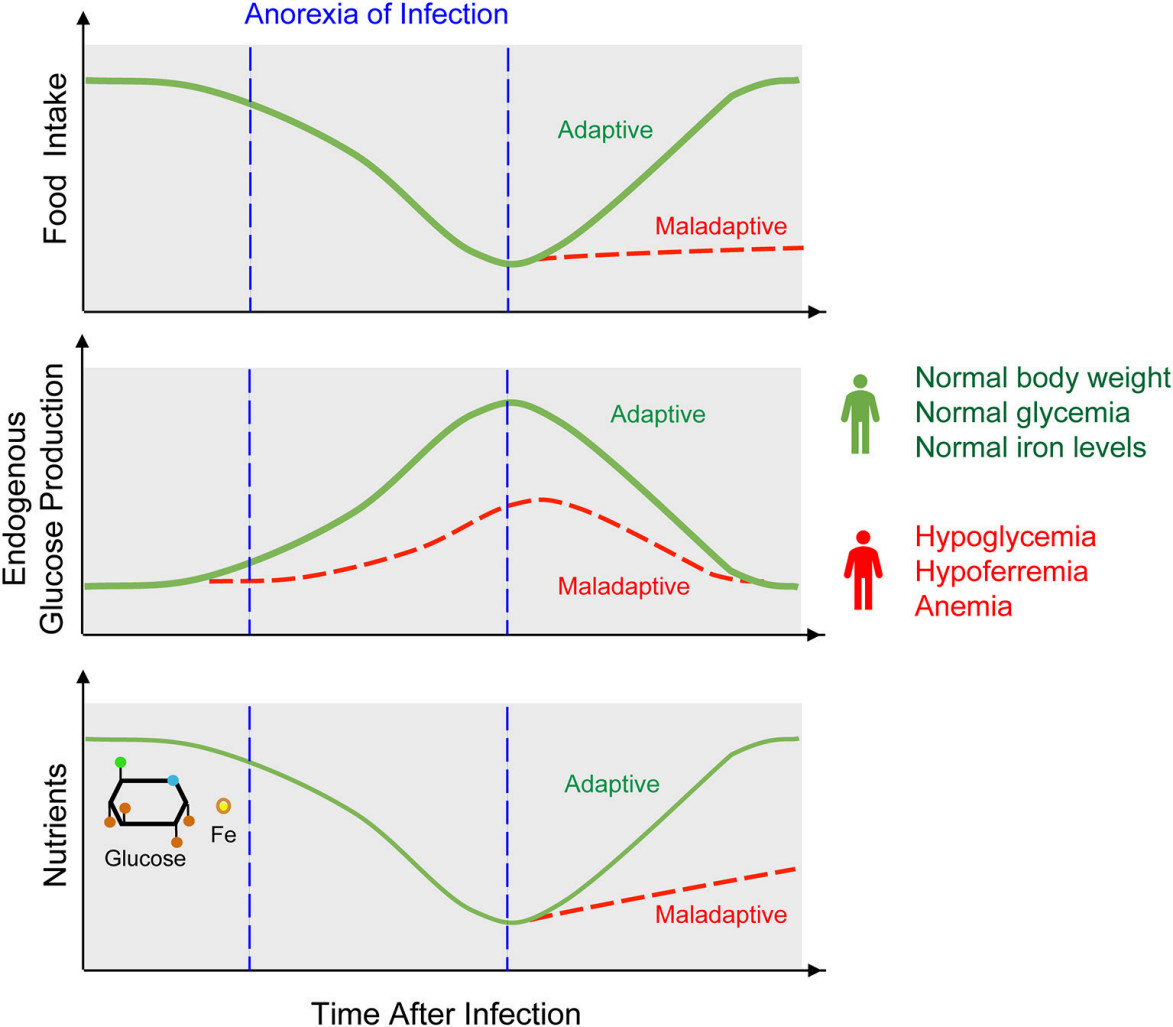
Anorexia of infection, metabolic adaptation, and outcome of infection. Anorexia is a hallmark of sickness behavior that consists on a transient reduction of food intake. Anorexia of infection probably limits pathogens from accessing nutrients, such as glucose or iron. This defense strategy however, cannot be sustained indefinitely as to avoid the development of hypoglycemia, hypoferremia, and anemia, eventually culminating in death of the infected host. Therefore, anorexia of infection must be coupled to a host metabolic response that regulates endogenous production of nutrients, such as illustrated for example for hepatic glucose production. This metabolic response is essential to establish disease tolerance to infection and may also impact on resistance to infection.

Infections can impose a distinctive host behavioral pattern referred to as sickness behavior (6, 7). This encompasses anorexia, characterized by a reduction of food intake, possibly aimed at limiting nutrient availability to invading pathogens (8, 9) (Figure 2). While protective against some classes of pathogens (10–12), anorexia of infection carries a high evolutionary trade-off in that nutrient deprivation can compromise host homeostasis. For example, reduced iron intake in response to infection can lead to anemia of chronic disease (13), while reduced glucose intake can lead to hypoglycemia (10, 14, 15). Here we explore how regulation of host iron and glucose metabolisms impact on the establishment of disease tolerance and possibly on resistance to infection.

## Iron metabolism and disease tolerance

Iron is the most abundant transition metal present on Earth and perhaps for this reason was co-opted early in evolution to catalyze vital redox-based reactions in most living organisms, from prokaryotes to eukaryotes (16). Like other divalent metals, iron can shift between reduced (ferrous; Fe^2+^) and oxidized (ferric; Fe^3+^) or even higher oxidation states (ferryl; Fe^4+^), via reversible exchange of electrons with electrophilic or nucleophilic molecules, respectively. In doing so, iron is at the center stage of a variety of vital biological processes, including the transport and storage of gaseous molecules, energy production, as well as other components of cellular metabolism (17, 18). Probably due to its essential role in supporting these vital functions, microbial pathogens evolved multiple strategies to acquire iron from their hosts, while infected hosts co-evolved to limit iron availability to pathogens (18–22). This evolutionarily conserved defense strategy against infection is referred to as nutritional immunity (23).

### Regulation of host iron metabolism in response to infection

Nutritional immunity is directed at inhibiting pathogens growth, via opposing mechanisms that limit nutrients' availability to intracellular or extracellular pathogens (18–22). Defense strategies limiting iron availability to intracellular pathogens rely on systemic inhibition of iron cellular import and can lead to hyperferremia (18–22). In contrast, defense strategies limiting iron availability to extracellular pathogens rely on cellular iron import mechanisms that promote cellular iron overload and hypoferremia (18–22). If uncontrolled, this can lead to the production of reactive oxygen species (ROS) via the Haber–Weiss–Fenton sequence (24), oxidizing and eventually damaging cellular macromolecules and organelles (22). In support of this notion, patients with genetic disorders characterized by cellular iron overload, such as hereditary hemochromatosis, are highly susceptible to a range of infections (25).

### Regulation of iron metabolism confers tissue damage control

Disruption of host iron homeostasis is a hallmark of many infectious diseases (18, 22), as illustrated for example in malaria, the disease caused by *Plasmodium* spp. infection (26–28), polymicrobial sepsis (14, 29), tuberculosis caused by *Mycobacterium tuberculosis* (30, 31) or acquired immune deficiency syndrome, caused by human immunodeficiency virus (HIV) infection (31). Regulation of host iron metabolism is critical to confer tissue damage control, and in doing so, establishes disease tolerance to infection, as demonstrated for example for malaria (32) or polymicrobial sepsis (14).

**Figure 3:**
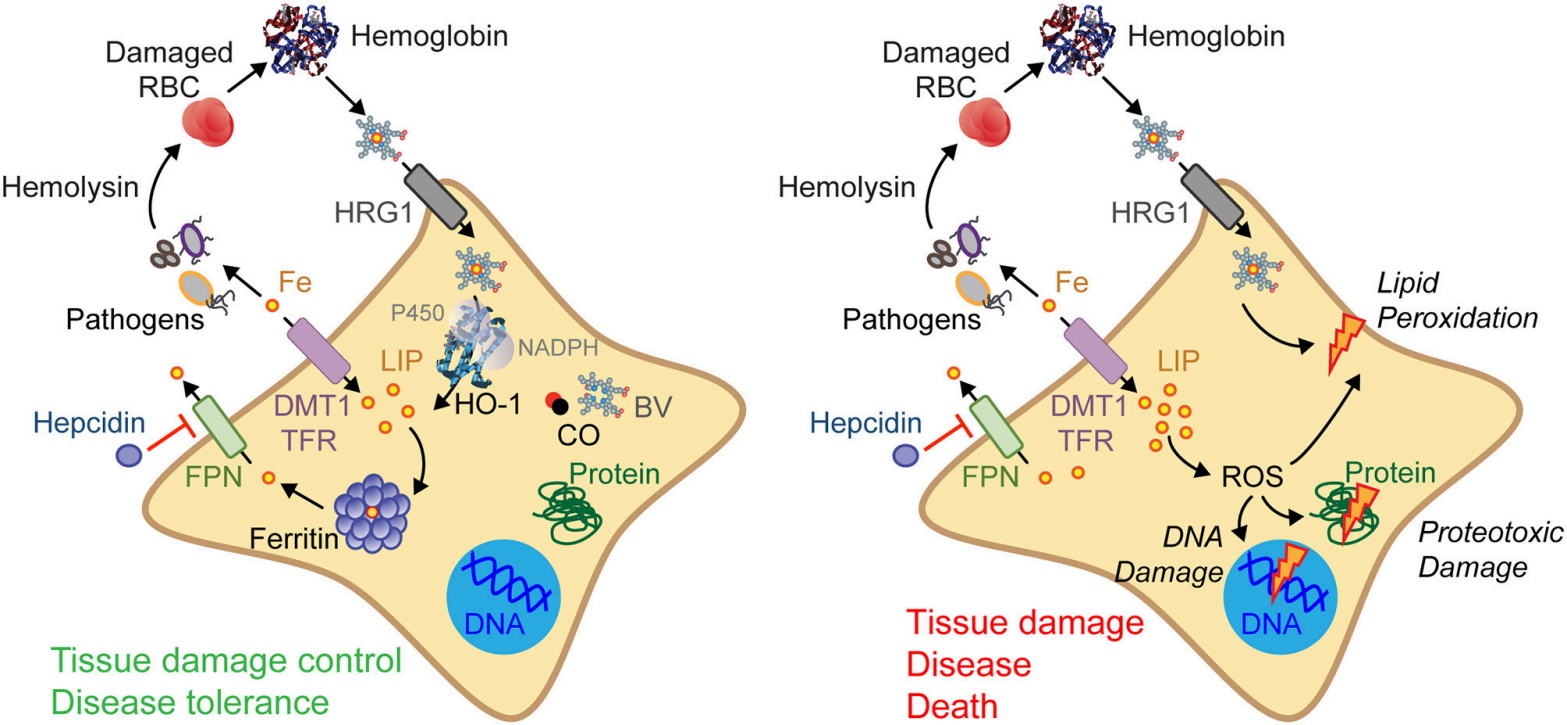
Regulation of cellular iron metabolism in response to infection. Several resistance mechanisms may be used to restrict extracellular pathogens from accessing iron. For example, host cells can import heme/iron via heme transporters, such as the heme responsive gene 1 (HRG1), or via iron transporters, such as the divalent metal transporter-1 (DMT1) or the transferrin (TF)-transferrin receptor (TFR) that uptakes iron-TF complexes. Intracellular heme is catabolized by HO-1, generating iron, biliverdin (BV), and carbon monoxide (CO) **(left)**. Hepcidin prevents cellular iron export via ferroportin (FPN) and as such LIP arising from heme catabolism must be stored by ferritin. These mechanisms are essential to confer tissue damage control and establish disease tolerance to systemic infections **(left)**. When these protective mechanisms fail **(right)** intracellular heme and LIP increases promoting the generation of ROS, damaging DNA, proteins, and lipids. Ultimately this can compromise tissue damage control and the establishment of disease tolerance to infection **(right)**.

The majority of the iron present in mammals exists in the form of heme (17, 33, 34), a tetrapyrrole ring that binds a central iron atom through different nitrogen atoms (34, 35). Heme is used essentially as a prosthetic group of hemoproteins, such as hemoglobin, myoglobin, or cytochrome c, where iron is deployed to exchange and store gaseous molecules or to transport electrons, respectively (33, 34). The largest pool of heme in mammals is found within hemoglobin in red blood cells (RBC), a prime target for invading pathogens in their search for iron (22, 33). As such, RBC lysis is a recurrent event associated with infection leading to the release of hemoglobin into plasma (17, 22, 36–38). Extracellular hemoglobin disassembles and auto-oxidizes, releasing its non-covalently bound prosthetic heme groups (33, 38) (Figure 3). This can lead to the generation of labile heme, that is, heme loosely bound to plasma acceptor proteins, macromolecules or low molecular weight ligands that fail to control its redox activity (36, 39). As it becomes bioavailable, a fraction of the labile heme in plasma acts in a pathogenic manner, compromising the establishment of disease tolerance to infection, as illustrated for malaria (38, 40, 41) or polymicrobial sepsis (14, 29).

Labile heme can also compromise resistance to infection via mechanisms inhibiting macrophage phagocytosis and impairing bacterial clearance (42) or mechanisms inducing macrophages to undergo programmed cell death (43). Moreover, labile heme can also be scavenged directly by bacterial pathogens, as demonstrated in the case of *Staphylococcus aureus* (44) or *Citrobacter rodentium* (45), promoting pathogen growth and compromising host resistance to infection (21, 46).

The pathological effects of labile heme are countered by host defense mechanisms that converge at the level of heme catabolism and storage of the iron extracted from heme (33, 34, 47). Under physiological conditions heme is catabolized by heme oxygenase-1 and -2 (HO-1 and HO-2), which cleave the tetrapyrrole ring, generating equimolar amounts of iron, carbon monoxide, and biliverdin (48). Upon infection, the stress-responsive HO-1 becomes the rate limiting enzyme in heme catabolism (33), playing a critical role in the establishment of disease tolerance to systemic infections, as illustrated for malaria (40, 41, 49) or polymicrobial sepsis (29).

The iron extracted via heme catabolism by heme oxygenases, integrates the cellular labile iron pool (LIP), becoming available to pathogens while catalyzing the production of ROS via the Haber–Weiss–Fenton sequence (24) (Figure 3). The pro-oxidant effects associated with excess heme catabolism and LIP overload are countered via the induction of cellular iron export by the solute carrier family 40 member 1 (SLC40A1), also known as ferroportin 1 (FPN1) (17, 22). Once excreted, iron is captured in plasma by transferrin (17, 22, 50) and delivered, via the transferrin receptor, to erythropoietic precursors where iron is required to support heme and hemoglobin synthesis (17, 22).

To prevent overt accumulation of extracellular iron, ferroportin expression and activity are downregulated by hepcidin, an acute-phase 25-amino acid peptide encoded by the *HAMP* gene (51, 52). In support of this notion, hepcidin accumulates in plasma in response to infection, inhibiting ferroportin expression/activity and impairing cellular iron export (51, 52). This can lead to cellular LIP accumulation, a potentially deleterious effect countered via iron storage and neutralization by ferritin (47, 53, 54).

Ferritin is a multimeric complex composed of ferritin heavy (heart) chain (FTH) and light (liver) chain (FTL) (47, 53, 54) (Figure 3). The ferroxidase activity of FTH converts pro-oxidant Fe^2+^ into nucleated Fe^3+^ (47, 53, 54), preventing LIP from participating in Haber–Weiss–Fenton sequence (24), limiting ROS generation and avoiding oxidative damage (17, 33). Supporting this notion, ferritin is essential to enforce tissue damage control and to establish disease tolerance to malaria (32) and to polymicrobial sepsis (14) (Figure 3). This protective effect depends on the ferroxidase activity of FTH, suggesting that iron conversion to its oxidized form (Fe^3+^) and subsequent incorporation into ferritin, are critical to establish disease tolerance to infection.

A significant proportion of ferritin is secreted (55), suggesting that the protective effects of ferritin are not restricted to its intracellular functions. In keeping with this notion, soluble ferritin is protective against *Escherichia coli* infection (56), and acts therapeutically to establish disease tolerance to polymicrobial sepsis (14). This argues for a role of extracellular ferritin as soluble iron chelator/transporter enforcing the establishment of disease tolerance to infection. Unexpectedly, the protective effects of ferritin extends beyond its antioxidant role, in that ferritin also controls glucose metabolism (14).

## Glucose metabolism and disease tolerance

Glucose is a key nutrient for most living organisms, acting both as a metabolic fuel for ATP production via glycolysis or mitochondrial electron transport and as a biosynthetic intermediate for amino acid, lipid, and nucleic acid synthesis (57). While glucose intake from food allows for systemic delivery, glucose can also be synthesized endogenously from glucose precursors via gluconeogenesis or glycogenolysis in the liver, kidneys, or intestine (58). Glucose uptake from diet and its endogenous synthesis are tightly regulated to maintain blood glucose levels within a homeostatic range (5, 59). Enforcing this homeostatic range is particularly challenging during an infection (5), given that pathogens and their hosts often compete for this nutrient. Similar to iron, the infected host evolved strategies to limit glucose availability to pathogens, while maintaining glucose levels within a range compatible with survival. One of the strategies limiting glucose availability to pathogens relies on reducing glucose and glucose precursors intake from diet, via anorexia of infection. This is probably a component of nutritional immunity conferring resistance against pathogens (8–12, 60) (Figure 2).

### Glucose availability in response to infection

The impact of anorexia of infection on the outcome of infectious diseases varies widely depending on the host and pathogen species (9–12, 61). In fruit flies, anorexia of infection promotes the establishment of disease tolerance to *Salmonella* Typhimurium infection, while compromising resistance to *Listeria monocytogenes* infection (11). In mice, anorexia of infection is protective against *L. monocytogenes* (10, 12), but deleterious against influenza virus infections (10, 62). Anorexia of infection also impacts on the outcome of gastrointestinal parasitic infections (60, 61), reducing body weight upon *Nippostrongylus brasiliensis* infection (63), while increasing immunopathology in response to *Trichostrongylus colubriformis* infection (64).

Mechanisms regulating anorexia of infection are not clearly established (9, 10, 61), but certainly encompass pathogen sensing via host pattern recognition receptors (PRR) (9). Signaling downstream of PRR elicits the production of interleukins (IL), such as IL-1, IL-6, IL-8, or tumor necrosis factor (TNF), which signal systemically to induce anorexia of infection as well as to regulate glucose metabolism (9). One of the mechanisms via which this occurs involves the secretion of leptin by adipose tissue (9, 65), an hormone that signals in the central nervous system (CNS) to reduce food intake and regulate energy consumption (66).

Pathogens can modulate anorexia of infection directly to promote their survival and/or transmission (9, 10, 61, 67). For example, *S*. Typhimurium inhibits PRR activation/signaling, reducing IL-1β secretion in the gut and increasing food consumption, as well as blood glucose levels (67). This reduces *S*. Typhimurium virulence and promotes host disease tolerance, while increasing *Salmonella* transmission (67), most likely as an evolutionary trade-off. The nematode *N. brasiliensis* also induces anorexia, via the regulation CNS signaling (68, 69), even though the exact mechanism by which this occurs has not been established.

Anorexia of infection is also associated with reduction in caloric intake, i.e., caloric restriction, which can *per se* modulate the outcome of infection (70). For example, caloric restriction increases susceptibility to polymicrobial (71) and viral infections (10, 72), while reducing *Plasmodium* virulence and promoting survival to malaria (73).

Although protective against bacterial infections (8, 9) mechanisms reducing blood glucose levels must be tightly regulated to prevent the development of lethal hypoglycemia. In support of this notion, inhibition of hepatic glucose production in mice carrying a liver-specific deletion of glucose 6 phosphatase 1 (*g6pc1*) compromises disease tolerance to polymicrobial infections (14). This suggests that while reducing blood glucose levels can be protective against bacterial infections (8, 9), endogenous glucose production is required to prevent the development of lethal hypoglycemia and establish disease tolerance to polymicrobial sepsis (14).

### Impact of metabolic diseases on infection

The impact of glucose metabolism on the outcome of infectious diseases is illustrated by the effect of metabolic diseases, such as obesity or diabetes, on the outcome of infections. For example, hyperglycemia in diabetic rodents is associated with increased susceptibility to polymicrobial sepsis (74, 75) as well to *L. monocytogenes* (76) or *M. tuberculosis* (77) infections. Moreover, hyperglycemia promotes intestinal permeability and increases susceptibility to bacterial infection in mice (78). This pathogenic effect is mediated via glucose import by intestinal epithelial cells, disrupting the functional integrity of the gut epithelium via a mechanism that interferes with epithelial tight and adherens junctions (78). Despite this experimental evidence, whether deregulation of glucose homeostasis impacts on the outcome of bacterial infections in humans remains unclear. For example, clinical evidence suggests that diabetes mellitus is not a major risk factor for sepsis severity (79), while both hyperglycemia and hypoglycemia are major risk factors for sepsis mortality (80, 81). Of note, rodents develop hypoglycemia rather than hyperglycemia in response to bacterial infections (14, 15, 82, 83). In some cases, hypoglycemia is preceded by a transient state of hyperglycemia, but whether this is triggered by infection and/or other associated experimental procedure is not clear (14, 15).

### Glucose control of innate and adaptive immune function

Regulation of host glucose metabolism can impact on pathogens directly or indirectly, via modulation of immune-driven resistance mechanisms (84–86). Proliferation, differentiation, and effector function of immune cells is regulated by two major metabolic programs, namely, oxidative phosphorylation, and aerobic glycolysis (84–86). Signaling via PRR in macrophages or dendritic cells shifts metabolic flux from oxidative phosphorylation to aerobic glycolysis, a phenomenon known as the Warburg effect (87). Despite being less energetically effective, glycolysis generates pyruvate, nicotinamide adenine dinucleotide (NADH), and other metabolic intermediates used by major biosynthetic pathways (84, 86). This metabolic shift also promotes the pentose phosphate pathway, generating nicotinamide adenine dinucleotide phosphate (NADPH), a critical component of the NADPH oxidase (NOX) enzyme complexes, generating ROS involved in pathogen killing (84, 86, 88). In contrast to their microbicidal effector functions, other macrophage effector functions promoting tissue healing and regeneration rely primarily on oxidative phosphorylation (86, 88).

A marked increase in aerobic glycolysis is also a hallmark of T cell activation, together with a more modest induction of oxidative phosphorylation (86, 89), presumably accommodating the reduction in oxygen availability that arises during infections (85, 90). This metabolic reprogramming is orchestrated by a complex mechanism involving the store-operated Ca^2+^ entry (SOCE), a key regulator of cellular calcium signaling (91), the hypoxia-inducible factor 1α (HIF1α), a transcriptional master regulator of hypoxia, as well as the mammalian target of rapamycin complex 1 (mTORC1), a master regulator of cell growth (84, 86, 92). Of note, mTORC1 controls the expression of glycolytic genes in innate and adaptive immune cells via a mechanism involving HIF1α (93–95). The relative impact of these metabolic pathways on the outcome of infections can be illustrated in the context of *M. tuberculosis* infection, where myeloid HIF1α plays a critical role to induce the Warburg effect (96), supporting resistance to *M. tuberculosis* (97). Similarly, mice lacking HIF1α in the myeloid compartment also fail to shift to aerobic glycolysis, succumbing to bacterial sepsis (98).

In contrast to effector T cells, memory T cells rely on oxidative phosphorylation to produce energy, using fatty acids to produce acetyl coenzyme A (acetyl-CoA) and fuel the Krebs cycle, via a mechanism known as fatty acid oxidation (FAO) (84–86). Moreover, recent work has shown that FAO in memory T cells, can occur not only via carnitine palmitoyltransferase IA (CPT IA)-dependent, but also independent mechanisms (99), suggesting that memory T cells are able to use a wide range of fatty acids in order to obtain energy. The switch between aerobic glycolysis and oxidative phosphorylation relies on a mechanism involving the transcription repressor Bcl-6 (100), which downregulates glycolytic genes and promotes the T and B cell differentiation toward the memory compartment (101–103). Presumably, the combined effect of reduced glycemia and Bcl-6 expression are likely to promote effector to memory T cell transition in response to infection. Whether glucose availability impacts on immune-driven resistance mechanisms remains, to the best of our knowledge, to be determined.

## Cross-talk between iron and glucose metabolism in response to infection

**Figure 4:**
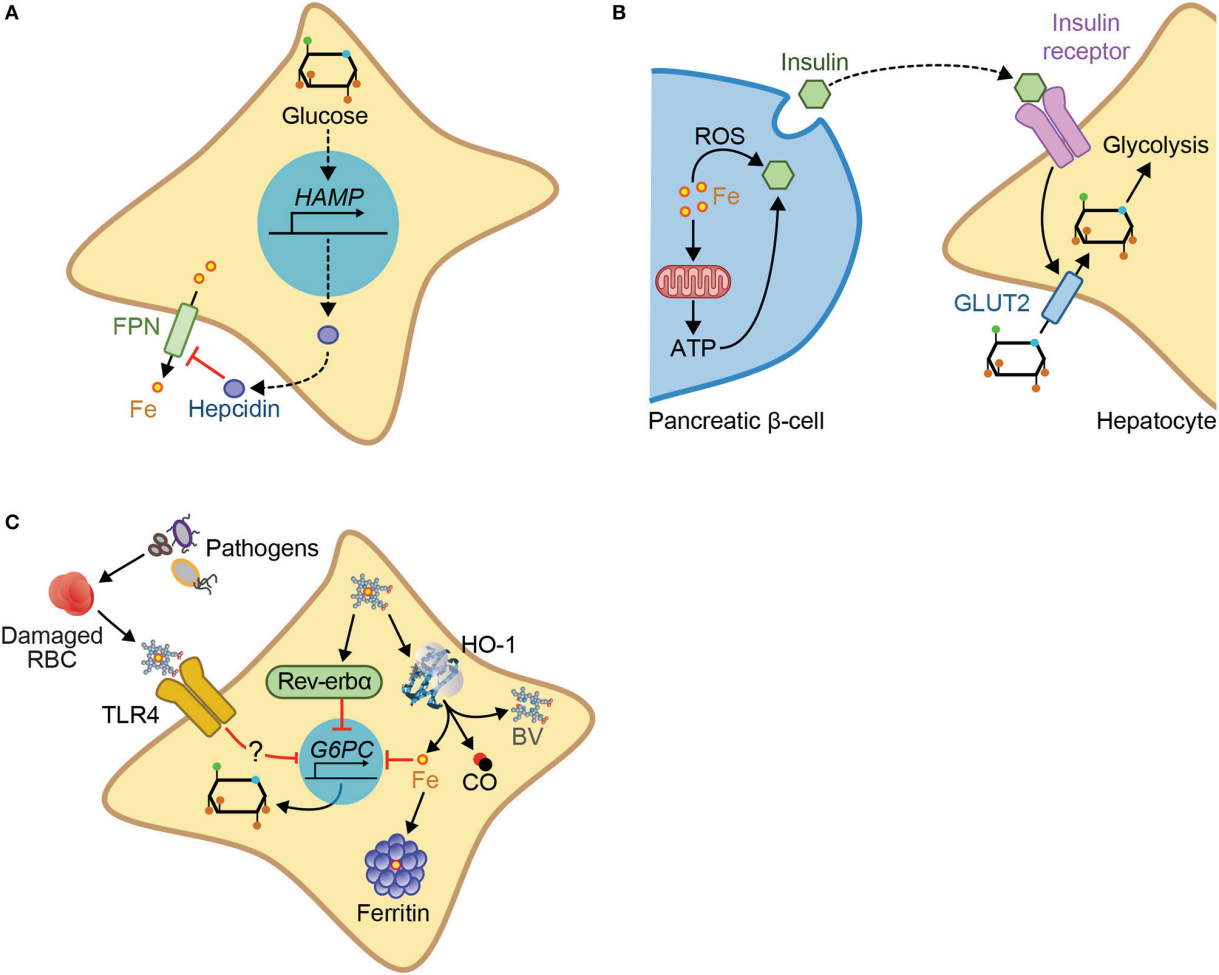
Mechanisms of iron-glucose metabolism cross-talk. Iron and glucose can cross-talk via different mechanisms: **(A)** Glucose increases expression of hepcidin, inhibiting cellular iron export via ferroportin. **(B)** Iron acts via the production of ROS or via mitochondrial respiration and subsequent ATP production, to promote insulin exocytosis by the pancreatic β-cells. Insulin binding to the insulin receptor in target cells, e.g., hepatocytes, promotes cellular glucose import via the glucose transporter 2 (GLUT2), and glycolysis. **(C)** In the context of infection, glucose metabolism can be regulated by heme via a pathway that involves TLR4, but which has not yet been fully described. Heme interaction with the nuclear receptor Rev-erbα, downregulates the transcription of gluconeogenic genes including *G6PC*, the enzyme catalyzing the last step of gluconeogenesis. *G6PC* is also downregulated by iron produced via heme catabolism by HO-1, an inhibitory effect countered by ferritin.

A functional interplay between iron and glucose metabolism has been established primarily in the context of metabolic diseases, such as porphyria (104, 105) or diabetes (106–108). For example, hepatic glucose production induces hepcidin expression (109, 110) (Figure 4A) and inhibits cellular iron export by ferroportin, leading to cellular iron overload and hypoferremia (110). Conversely, cellular iron overload regulates insulin production in pancreatic β-cells (106, 111) and is thought to contribute critically to impair glucose metabolism in diabetic patients (112, 113) (Figure 4B). This interplay is probably operational in other pathologic conditions such as atherosclerosis (107) or β-thalassemia major (114).

More recently a crosstalk between iron and glucose metabolism has also been established in the context of infections (14, 115, 116). Namely, iron intake from diet leads to decreased pathogen virulence, without interfering with pathogen burden, favoring asymptomatic infection with the enteric pathogen *C. rodentium* (116). This occurs through a mechanism via which iron intake promotes insulin resistance, reducing glucose uptake by the intestine, and thus promoting glucose availability in the gut, leading to the suppression of virulence factors (116). Deregulation of host iron metabolism in response to polymicrobial infection compromises the establishment of disease tolerance to sepsis, via a mechanism that deregulates glucose metabolism (14, 117), thus also illustrating the crosstalk between iron and glucose. This pathologic mechanism is driven by labile heme, which plays a central role in the pathogenesis of sepsis (29). Namely, labile heme inhibits hepatic G6pase and consequently glucose production leading to hypoglycemia (14) (Figure 4C). This pathogenic effect has been linked functionally to a transcriptional repression of *g6pc1* gene (14). In support of this notion, mice lacking hepatic *g6pc1* develop lethal hypoglycemia in response to polymicrobial sepsis or heme administration (14). This suggests that hepatic glucose production is required to counter the hypoglycemia induced by labile heme (14). This is also consistent with the notion that deregulation of glucose metabolism plays a central role in pathogenesis of infectious diseases, including sepsis (10, 14, 80, 81, 118). This occurs via a mechanism that is not associated with modulation of host pathogen load (10, 14, 117), demonstrating that regulation of glucose metabolism controls the establishment of disease tolerance to infection (10, 14, 117).

The molecular mechanism via which labile heme induces hypoglycemia is not entirely clear but has been linked to signaling via Toll-like receptor 4 (TLR4) (14) (Figure 4C), a PRR that senses labile heme (119). This is consistent with the induction of hypoglycemia by TLR4 ligands, such as LPS (120). Whether heme sensing by TLR4 mediates the development of hypoglycemia during polymicrobial sepsis was not established. The pathway through which heme represses *g6pc1* transcription (14, 121) is likely to involve the heme sensor and transcriptional repressor Rev-erbα (121) (Figure 4C). Whether this mechanism is operational *in vivo* to repress hepatic glucose production and elicit hypoglycemia in response to infection remains to be established. It is possible as well that TLR4 and Rev-erbα synergize to repress *g6pc1* transcription in hepatocytes.

Iron sequestration by ferritin counters heme-driven repression of *g6pc1* transcription, suggesting that heme represses *g6pc1* transcription via a mechanism involving iron (14). In keeping with this notion, polymicrobial infections in mice are associated with the induction of ferritin in the liver, which is essential to sustain hepatic glucose-6-phosphatase (G6Pase) expression and counter the development of lethal hypoglycemia (14, 122) (Figure 4C). Whether iron accumulation in hepatocytes synergizes with TLR4 and Rev-erbα to repress *g6pc1* transcription remains to be established.

Regulation of hepatic glucose production by ferritin may be part of an adaptive response promoting the development of insulin resistance, presumably countering unfettered cellular glucose utilization in host tissues and allowing to restore normal blood glucose levels (123). This effect of ferritin should also contribute to prevent the development of hypoglycemia in response to infections.

## Conclusions and future perpectives

Resistance to infection is generally perceived as the predominant host defense strategy against infection. This dogma has been challenged by the recurrent observation that the severity of infectious diseases can at times be dissociated from host pathogen burden. In the last few years these observations have been interpreted as revealing disease tolerance as a critical host defense strategy against infection. This defense strategy relies on tissue damage control mechanisms controlling the metabolic output of host tissue and maintaining vital homeostatic parameters within a range compatible with host survival. This is illustrated for mechanisms regulating iron and glucose metabolism, which cross-talk to establish disease tolerance to infection. To what extent these tissue damage control mechanisms may be targeted therapeutically remains to be established.

## Author contributions

ARC wrote the manuscript with SW and MPS. All authors listed have made a substantial, direct and intellectual contribution to the work, and approved it for publication.

### Conflict of interest statement

The authors declare that the research was conducted in the absence of any commercial or financial relationships that could be construed as a potential conflict of interest.

## References

[1] Medzhitov R Schneider DS Soares MP. Disease tolerance as a defense strategy. Science (2012) 335:335–41. 10.1126/science.1214935 22363001 PMC3564547

[2] Schneider DS Ayres JS. Two ways to survive infection: what resistance and tolerance can teach us about treating infectious diseases. Nat Rev Immunol. (2008) 8:8–95. 10.1038/nri2432 18927577 PMC4368196

[3] Soares MP Gozzelino R Weis S. Tissue damage control in disease tolerance. Trends Immunol. (2014) 35:35–94. 10.1016/j.it.2014.08.001 25182198

[4] Chovatiya R Medzhitov R. Stress, inflammation, and defense of homeostasis. Mol Cell (2014) 54:54–8. 10.1016/j.molcel.2014.03.030 24766892 PMC4048989

[5] Kotas ME Medzhitov R. Homeostasis, inflammation, and disease susceptibility. Cell (2015) 160:160–27. 10.1016/j.cell.2015.02.010 25723161 PMC4369762

[6] Hart BL. Biological basis of the behavior of sick animals. Neurosci Biobehav Rev. (1988) 12:12–37. 10.1016/S0149-7634(88)80004-6 3050629

[7] Dantzer R O'Connor JC Freund GG Johnson RW Kelley KW. From inflammation to sickness and depression: when the immune system subjugates the brain. Nat Rev Neurosci. (2008) 9:9–56. 10.1038/nrn2297 18073775 PMC2919277

[8] Murray MJ Murray AB. Anorexia of infection as a mechanism of host defense. Am J Clin Nutr. (1979) 32:32–6. 10.1093/ajcn/32.3.593 283688

[9] Langhans W. Anorexia of infection: current prospects. Nutrition (2000) 16:16–1005. 10.1016/S0899-9007(00)00421-4 11054606

[10] Wang A Huen SC Luan HH Yu S Zhang C Gallezot JD. Opposing effects of fasting metabolism on tissue tolerance in bacterial and viral inflammation. Cell (2016) 166:166–25 e12. 10.1016/j.cell.2016.07.026 27610573 PMC5555589

[11] Ayres JS Schneider DS. The role of anorexia in resistance and tolerance to infections in Drosophila. PLoS Biol. (2009) 7:7. 10.1371/journal.pbio.1000150 19597539 PMC2701602

[12] Wing EJ Young JB. Acute starvation protects mice against Listeria monocytogenes. Infect Immun. (1980) 28:28–6. 6772566 10.1128/iai.28.3.771-776.1980 PMC551017

[13] Weiss G Goodnough LT. Anemia of chronic disease. N Engl J Med. (2005) 352:352–23. 10.1056/NEJMra041809 15758012

[14] Weis S Carlos AR Moita MR Singh S Blankenhaus B Cardoso S. Metabolic adaptation establishes disease tolerance to sepsis. Cell (2017) 169:169–75.e14. 10.1016/j.cell.2017.05.031 28622511 PMC5480394

[15] Ferreira FBD dos Santos C Bruxel MA Nunes EA Spiller F Rafacho A. Glucose homeostasis in two degrees of sepsis lethality induced by caecum ligation and puncture in mice. Int J Exp Pathol. (2017) 98:98–40. 10.1111/iep.12255 29226508 PMC5826970

[16] Andrews NC Schmidt PJ. Iron homeostasis. Annu Rev Physiol. (2007) 69:69–85. 10.1146/annurev.physiol.69.031905.164337 17014365

[17] Muckenthaler MU Rivella S Hentze MW Galy B. A red carpet for iron metabolism. Cell (2017) 168:168–61. 10.1016/j.cell.2016.12.034 28129536 PMC5706455

[18] Ganz T Nemeth E. Iron homeostasis in host defence and inflammation. Nat Rev Immunol. (2015) 15:15–10. 10.1038/nri3863 26160612 PMC4801113

[19] Palmer LD Skaar EP. Transition metals and virulence in bacteria. Annu Rev Genet. (2016) 50:50–91. 10.1146/annurev-genet-120215-035146 27617971 PMC5125913

[20] Hood MI Skaar EP. Nutritional immunity: transition metals at the pathogen–host interface. Nat Rev Microbiol. (2012) 10:10–37. 10.1038/nrmicro2836 22796883 PMC3875331

[21] Nuñez G Sakamoto K Soares MP Núñez G Sakamoto K Soares MP. Innate Nutritional Immunity. J Immunol. (2018): 11–8. 10.4049/jimmunol.1800325 29914937 PMC6028930

[22] Soares MP Weiss G. The Iron age of host-microbe interactions. EMBO Rep. (2015) 16:16–500. 10.15252/embr.201540558 26474900 PMC4641501

[23] Weinberg ED. Nutritional immunity. host's attempt to withold iron from microbial invaders. JAMA (1975) 231:231. 1243565 10.1001/jama.231.1.39

[24] Aisen P Enns C Wessling-Resnick M. Chemistry and biology of eukaryotic iron metabolism. Int J Biochem Cell Biol. (2001) 33:33–59. 10.1016/S1357-2725(01)00063-2 11470229

[25] Khan FA Fisher MA Khakoo RA. Association of hemochromatosis with infectious diseases: expanding spectrum. Int J Infect Dis. (2007) 11:11–7. 10.1016/j.ijid.2007.04.007 17600748

[26] Prentice AM Verhoef H Cerami C. Iron fortification and malaria risk in children. JAMA (2013) 310:310–5. 10.1001/jama.2013.6771 24002276 PMC6136145

[27] Mabeza GF Loyevsky M Gordeuk VR Weiss G. Iron chelation therapy for malaria: a review. Pharmacol Ther. (1999) 81:81–75. 10.1016/S0163-7258(98)00037-0 10051178

[28] Portugal S Drakesmith H Mota MM. Superinfection in malaria: plasmodium shows its iron will. EMBO Rep. (2011) 12:12–42. 10.1038/embor.2011.213 22081142 PMC3245699

[29] Larsen R Gozzelino R Jeney V Tokaji L Bozza FA Japiassu AM. A central role for free heme in the pathogenesis of severe sepsis. Sci Transl Med. (2010) 2:2ra71. 10.1126/scitranslmed.3001118 20881280

[30] Banerjee S Farhana A Ehtesham NZ Hasnain SE. Iron acquisition, assimilation and regulation in mycobacteria. Infect Genet Evol. (2011) 11:11–38. 10.1016/j.meegid.2011.02.016 21414421

[31] McDermid JM Hennig BJ van der Sande M Hill AV Whittle HC Jaye A. Host iron redistribution as a risk factor for incident tuberculosis in HIV infection: an 11-year retrospective cohort study. BMC Infect Dis. (2013) 13:13. 10.1186/1471-2334-13-48 23360117 PMC3568026

[32] Gozzelino R Andrade BB Larsen R Luz NF Vanoaica L Seixas E. Metabolic adaptation to tissue iron overload confers tolerance to malaria. Cell Host Microbe (2012) 12:12–704. 10.1016/j.chom.2012.10.011 23159058

[33] Gozzelino R Jeney V Soares MP. Mechanisms of cell protection by heme oxygenase-1. Annu Rev Pharmacol Toxicol. (2010) 50:50–54. 10.1146/annurev.pharmtox.010909.105600 20055707

[34] Tsiftsoglou AS Tsamadou AI Papadopoulou LC. Heme as key regulator of major mammalian cellular functions: molecular, cellular, and pharmacological aspects. Pharmacol Ther. (2006) 111:111–45. 10.1016/j.pharmthera.2005.10.017 16513178

[35] Poulos TL. The Janus nature of heme. Nat Prod Rep. (2007) 24:24. 10.1039/b604195g 17534526

[36] Soares MP Bozza MT. Red alert: labile heme is an alarmin. Curr Opin Immunol. (2016) 38:38–100. 10.1016/j.coi.2015.11.006 26741528

[37] Soares MP Hamza I. Macrophages and Iron metabolism. Immunity (2016) 44:44–504. 10.1016/j.immuni.2016.02.016 26982356 PMC4794998

[38] Ferreira A Balla J Jeney V Balla G Soares MP A central role for free heme in the pathogenesis of severe malaria: the missing link? J Mol Med. (2008) 86:86–111. 10.1007/s00109-008-0368-5 18641963

[39] Gouveia Z Carlos AR Yuan X Aires-da-Silva F Stocker R Maghzal GJ. Characterization of plasma labile heme in hemolytic conditions. FEBS J. (2017) 284:284–301. 10.1111/febs.14192 28783254 PMC5978748

[40] Ferreira A Marguti I Bechmann I Jeney V Chora A Palha NR. Sickle hemoglobin confers tolerance to plasmodium infection. Cell (2011) 145:145–409. 10.1016/j.cell.2011.03.049 21529713

[41] Pamplona A Ferreira A Balla J Jeney V Balla G Epiphanio S. Heme oxygenase-1 and carbon monoxide suppress the pathogenesis of experimental cerebral malaria. Nat Med. (2007) 13:13–10. 10.1038/nm1586 17496899

[42] Martins R Maier J Gorki A-D Huber KVM Sharif O Starkl P. Heme drives hemolysis-induced susceptibility to infection via disruption of phagocyte functions. Nat Immunol. (2016) 17:17–72. 10.1038/ni.3590 27798618

[43] Fortes GB Alves LS de Oliveira R Dutra FF Rodrigues D Fernandez PL. Heme induces programmed necrosis on macrophages through autocrine TNF and ROS production. Blood (2012) 119:119–75. 10.1182/blood-2011-08-375303 22262768 PMC3358230

[44] Skaar EP Humayun M Bae T DeBord KL Schneewind O. Iron-Source preference of staphylococcus aureus infections. Science (2004) 305:305–8. 10.1126/science.1099930 15361626

[45] Sakamoto K Kim Y-G Hara H Kamada N Caballero-Flores G Tolosano E. IL-22 controls iron-dependent nutritional immunity against systemic bacterial infections. Sci Immunol. (2017) 2:2eaai8371. 10.1126/sciimmunol.aai8371 28286877 PMC5345941

[46] Martins R Knapp S Heme and hemolysis in innate immunity: adding insult to injury. Curr Opin Immunol. (2018) 50:50–20. 10.1016/j.coi.2017.10.005 29107115

[47] Gozzelino R Soares MP. Coupling heme and iron metabolism via ferritin H chain. Antioxid Redox Signal. (2014) 20:20–69. 10.1089/ars.2013.5666 24124891 PMC3961798

[48] Larsen R Gouveia Z Soares MP Gozzelino R. Heme cytotoxicity and the pathogenesis of immune-mediated inflammatory diseases. Front Pharmacol. (2012) 3:3. 10.3389/fphar.2012.00077 22586395 PMC3343703

[49] Seixas E Gozzelino R Chora A Ferreira A Silva G Larsen R. Heme oxygenase-1 affords protection against noncerebral forms of severe malaria. Proc Natl Acad Sci USA. (2009) 106:106–42. 10.1073/pnas.0903419106 19706490 PMC2728109

[50] Lane DJ Merlot AM Huang ML Bae DH Jansson PJ Sahni S Cellular iron uptake, trafficking and metabolism: key molecules and mechanisms and their roles in disease. Biochim Biophys Acta (2015) 1853:1853–44. 10.1016/j.bbamcr.2015.01.021 25661197

[51] Nemeth E Tuttle MS Powelson J Vaughn MB Donovan A Ward DM. Hepcidin regulates cellular iron efflux by binding to ferroportin and inducing its internalization. Science (2004) 306:306–3. 10.1126/science.1104742 15514116

[52] Drakesmith H Prentice AM. Hepcidin and the iron-infection axis. Science (2012) 338:338–72. 10.1126/science.1224577 23139325

[53] Harrison PM Arosio P The ferritins: molecular properties, iron storage function and cellular regulation. Biochim Biophys Acta (1996) 1275:1275–203. 10.1016/0005-2728(96)00022-9 8695634

[54] Arosio P Elia L Poli M. Ferritin, cellular iron storage and regulation. IUBMB Life (2017) 69:69–22. 10.1002/iub.1621 28349628

[55] Meyron-Holtz EG Moshe-Belizowski S Cohen LA. A possible role for secreted ferritin in tissue iron distribution. J Neural Transm. (2011) 118:118–47. 10.1007/s00702-011-0582-0 21298454

[56] Lipinski P Jarzabek Z Broniek S Zagulski T. Protective effect of tissue ferritins in experimental *Escherichia coli* infection of mice *in vivo*. Int J Exp Pathol. (1991) 72:72–30. 1768608 PMC2002450

[57] Navdeep C Navigating Metabolism Press. 1st ed. Cold Spring Harbor :Laboratory Press (2015).

[58] Soty M Gautier-Stein A Rajas F Mithieux G. Gut-Brain glucose signaling in energy homeostasis. Cell Metab. (2017) 25:25–42. 10.1016/j.cmet.2017.04.032 28591631

[59] Soty M Penhoat A Amigo-Correig M Vinera J Sardella A Vullin-Bouilloux F. A gut–brain neural circuit controlled by intestinal gluconeogenesis is crucial in metabolic health. Mol Metab. (2015) 4:4–17. 10.1016/j.molmet.2014.12.009 25685698 PMC4314540

[60] Kyriazakis II Tolkamp BJ Hutchings MR. Towards a functional explanation for the occurrence of anorexia during parasitic infections. Anim Behav. (1998) 56:56–74. 10.1006/anbe.1998.0761 9787017

[61] Colditz IG. Six costs of immunity to gastrointestinal nematode infections. Parasite Immunol. (2008) 30:30–70. 10.1111/j.1365-3024.2007.00964.x 18186766

[62] Swiergiel AH Smagin GN Dunn AJ Influenza virus infection of mice induces anorexia: comparison with endotoxin and interleukin-1 and the effects of indomethacin. Pharmacol Biochem Behav. (1997) 57:57–96. 10.1016/S0091-3057(96)00335-8 9164599

[63] Crompton DWT Walters DE Arnold S. Changes in the food intake and body weight of protein-malnourished rats infected with *Nippostrongylus brasiliensis* (*Nematoda*). Parasitology (1981) 82:82–38. 10.1017/S0031182000041834 7208102

[64] Greer AW Stankiewicz M Jay NP McAnulty RW Sykes AR The effect of concurrent corticosteroid induced immuno-suppression and infection with the intestinal parasite *Trichostrongylus colubriformis* on food intake and utilization in both immunologically naïve and competent sheep. Anim Sci. (2005) 80:80–99. 10.1079/ASC41100089

[65] Sachot C Poole S Luheshi GN. Circulating leptin mediates lipopolysaccharide-induced anorexia and fever in rats. J Physiol. (2004) 561:561–72. 10.1113/jphysiol.2004.074351 15388782 PMC1665347

[66] Zhang Y Proenca R Maffei M Barone M Leopold L Friedman JM. Positional cloning of the mouse obese gene and its human homologue. Nature (1994) 372:372–32. 10.1038/372425a0 7984236

[67] Rao S Schieber AM O'Connor CP Leblanc M Michel D Ayres JS. Pathogen-Mediated inhibition of anorexia promotes host survival and transmission. Cell (2017) 168:168–16 e12. 10.1016/j.cell.2017.01.006 28129542 PMC5324724

[68] Horbury SR Mercer JG Chappell LH. Anorexia induced by the parasitic nematode, *Nippostrongylus brasiliensis*: effects on NPY and CRF gene expression in the rat hypothalamus. J Endocrinol. (1995) 7:7–73. 10.1111/j.1365-2826.1995.tb00728.x 8748124

[69] Roberts HC Hardie LJ Chappell LH Mercer JG Parasite-induced anorexia: leptin, insulin and corticosterone responses to infection with the nematode, *Nippostrongylus brasiliensis*. Parasitology (1999) 117–23. 10.1017/S0031182098003503 10070669

[70] Speakman JR Mitchell SE. Caloric restriction. Mol Aspects Med. (2011) 32:32–221. 10.1016/j.mam.2011.07.001 21840335

[71] Sun D Muthukumar AR Lawrence RA Fernandes G. Effects of calorie restriction on polymicrobial peritonitis induced by cecum ligation and puncture in young C57BL/6 mice. Clin Diagn Lab Immunol. (2001) 8:8–11. 10.1128/CDLI.8.5.1003-1011.2001 11527818 PMC96186

[72] Ritz BW Aktan I Nogusa S Gardner EM. Energy restriction impairs natural killer cell function and increases the severity of influenza infection in young adult male C57BL/6 mice. J Nutr. (2008) 138:138–75. 10.3945/jn.108.093633 18936230 PMC2635521

[73] Mancio-Silva L Slavic K Grilo Ruivo MT Grosso AR Modrzynska KK Vera IM. Nutrient sensing modulates malaria parasite virulence. Nature (2017) 547:547–6. 10.1038/nature23009 28678779 PMC5511512

[74] Filgueiras LR Jr Martins JO Serezani CH Capelozzi VL Montes MBA Jancar S. Sepsis-induced Acute Lung Injury (ALI) is milder in diabetic rats and correlates with impaired NFkB activation. PLoS ONE (2012) 7:7. 10.1371/journal.pone.0044987 23024779 PMC3443211

[75] Spiller F Carlos D Souto FO de Freitas A Soares FS Vieira SM. α1-Acid glycoprotein decreases neutrophil migration and increases susceptibility to sepsis in diabetic mice. Diabetes (2012) 61:61–91. 10.2337/db11-0825 22415874 PMC3357278

[76] Ikejima S Sasaki S Sashinami H Mori F Ogawa Y Nakamura T. Impairment of host resistance to Listeria monocytogenes infection in liver of db/db and ob/ob mice. Diabetes (2005) 54:54–9. 10.2337/diabetes.54.1.182 15616027

[77] Martens GW Arikan MC Lee J Ren F Greiner D Kornfeld H. Tuberculosis susceptibility of diabetic mice. Am J Respir Cell Mol Biol. (2007) 37:37–24. 10.1165/rcmb.2006-0478OC 17585110 PMC2048677

[78] Winer DA Luck H Tsai S Winer S. The intestinal immune system in obesity and insulin resistance. Cell Metab. (2016) 23:23–26. 10.1016/j.cmet.2016.01.003 26853748

[79] van Vught LA Scicluna BP Hoogendijk AJ Wiewel MA Klein Klouwenberg PMC Cremer OL Association of diabetes and diabetes treatment with the host response in critically ill sepsis patients. Crit Care (2016) 20:20 10.1186/s13054-016-6571429-8 27495247 PMC4975896

[80] Miller SI Wallace RJ Musher DM Septimus EJ Kohl S Baughn RE. Hypoglycemia as a manifestation of sepsis. Am J Med. (1980) 68:68–54. 10.1016/0002-9343(80)90250-8 6990758

[81] Van Cromphaut SJ Vanhorebeek I Van den Berghe G Berghe G Glucose metabolism and insulin resistance in sepsis. Curr Pharm Des. (2008) 14:14–99. 10.2174/138161208784980563 18691100

[82] Heuer JG Bailey DL Sharma GR Zhang T Ding C Ford A Cecal ligation and puncture with total parenteral nutrition: a clinically relevant model of the metabolic, hormonal, and inflammatory dysfunction associated with critical illness. J Surg Res. (2004) 121:121–86. 10.1016/j.jss.2004.04.018 15501457

[83] Singamsetty S Shah FA Guo L Watanabe Y McDonald S Sharma R Zhang Y. Early initiation of low-level parenteral dextrose induces an accelerated diabetic phenotype in septic C57BL/6J mice. Appl Physiol Nutr Metab. (2016) 41:41–9. 10.1139/apnm-2015-0213 26624964 PMC4756584

[84] Buck MD Sowell RT Kaech SM Pearce EL. Metabolic instruction of immunity. Cell (2017) 169:169–86. 10.1016/j.cell.2017.04.004 28475890 PMC5648021

[85] Loftus RM Finlay DK Immunometabolism: cellular metabolism turns immune regulator. J Biol Chem. (2016) 291:291–10. 10.1074/jbc.R115.693903 26534957 PMC4697146

[86] O'Neill LAJ Kishton RJ Rathmell J. A guide to immunometabolism for immunologists. Nat Rev Immunol. (2016) 16:16–65. 10.1038/nri.2016.70 27396447 PMC5001910

[87] Warburg O. On the origin of cancer cells. Science (1956) 123:123–14. 10.1126/science.123.3191.309 13298683

[88] Kelly B O'Neill LA. Metabolic reprogramming in macrophages and dendritic cells in innate immunity. Cell Res. (2015) 25:25–84. 10.1038/cr.2015.68 26045163 PMC4493277

[89] Pearce EL Poffenberger MC Chang C-HH Jones RG Aghajanirefah A Matarese F Fueling immunity: insights into metabolism and lymphocyte function. Science (2013) 342:342 10.1126/science.1242454 PMC4486656 24115444

[90] Donnelly RP Finlay DK. Glucose, glycolysis and lymphocyte responses. Mol Immunol. (2015) 68:68–9. 10.1016/j.molimm.2015.07.034 26260211

[91] Vaeth M Maus M Klein-Hessling S Freinkman E Yang J Eckstein M. Store-operated Ca2+ entry controls clonal expansion of T cells through metabolic reprogramming. Immunity (2017) 47:47–79.e6. 10.1016/j.immuni.2017.09.003 29030115 PMC5683398

[92] Saxton RA Sabatini DM. mTOR signaling in growth, metabolism, and disease. Cell (2017) 168:168–76. 10.1016/j.cell.2017.02.004 28283069 PMC5394987

[93] Finlay DK Rosenzweig E Sinclair L V Feijoo-Carnero C Hukelmann JL Rolf J. PDK1 regulation of mTOR and hypoxia-inducible factor 1 integrate metabolism and migration of CD8+ T cells. J Exp Med. (2012) 209:209–53. 10.1084/jem.20112607 23183047 PMC3526360

[94] Shi LZ Wang R Huang G Vogel P Neale G Green DR. HIF1alpha-dependent glycolytic pathway orchestrates a metabolic checkpoint for the differentiation of TH17 and Treg cells. J Exp Med. (2011) 208:208–76. 10.1084/jem.20110278 21708926 PMC3135370

[95] Cramer T Yamanishi Y Clausen BE Förster I Pawlinski R Mackman N. HIF-1α Is essential for myeloid cell-mediated inflammation. Cell (2003) 112:112 645–57. 10.1016/S0092-8674(03)00154-5 12628185 PMC4480774

[96] Shi L Salamon H Eugenin EA Pine R Cooper A Gennaro ML. Infection with Mycobacterium tuberculosis induces the Warburg effect in mouse lungs. Sci Rep. (2015) 5:5. 10.1038/srep18176 26658723 PMC4674750

[97] Braverman J Sogi KM Benjamin D Nomura DK Stanley SA. HIF-1α Is an essential mediator of IFN-γ-dependent immunity to mycobacterium tuberculosis. J Immunol. (2016) 197:197–97. 10.4049/jimmunol.1600266 27430718 PMC4976004

[98] Cheng S-C Quintin J Cramer RA Shepardson KM Saeed S Kumar V mTOR- and HIF-1 -mediated aerobic glycolysis as metabolic basis for trained immunity. Science (2014) 345:345 10.1126/science.1250684 PMC4226238 25258083

[99] Raud B Roy DG Divakaruni AS Tarasenko TN Franke R Ma EH. Etomoxir actions on regulatory and memory T cells are independent of Cpt1a-mediated fatty acid oxidation. Cell Metab. (2018) 28:28–15.e7. 10.1016/j.cmet.2018.06.002 30043753 PMC6747686

[100] Oestreich KJ Read KA Gilbertson SE Hough KP McDonald PW Krishnamoorthy V. Bcl-6 directly represses the gene program of the glycolysis pathway. Nat Immunol. (2014) 15:15–64. 10.1038/ni.2985 25194422 PMC4226759

[101] Pepper M Pagan AJ Igyarto BZ Taylor JJ Jenkins MK. Opposing signals from the Bcl6 transcription factor and the interleukin-2 receptor generate T helper 1 central and effector memory cells. Immunity (2011) 35:35–95. 10.1016/j.immuni.2011.09.009 22018468 PMC3208313

[102] Crotty S Johnston RJ Schoenberger SP Effectors and memories: Bcl-6 and Blimp-1 in T and B lymphocyte differentiation. Nat Immunol. (2010) 11:11–20. 10.1038/ni.1837 PMC2864556 20084069

[103] Ichii H Sakamoto A Hatano M Okada S Toyama H Taki S. Role for Bcl-6 in the generation and maintenance of memory CD8+ T cells. Nat Immunol. (2002) 3:3–63. 10.1038/ni802 12021781

[104] Khadilkar S V Yadav RS Patel BA Porphyrias. In: Neuromuscular Disorders. Singapore: Springer Singapore (2018). p. 493–502.

[105] Telega GW Metabolic and genetic liver diseases: porphyrias. In: Saeian K, Shaker R, editors. Liver Disorders. Cham: Springer (2017). p. 381–7.

[106] Fernandez-Real JM McClain D Manco M. Mechanisms linking glucose homeostasis and iron metabolism toward the onset and progression of type 2 diabetes. Diabetes Care (2015) 38:38–76. 10.2337/dc14-3082 26494808

[107] Fernandez-Real JM Manco M. Effects of iron overload on chronic metabolic diseases. Lancet Diabetes Endocrinol. (2014) 2:2–26. 10.1016/S2213-8587(13)70174-8 24731656

[108] Simcox JA McClain DA. Iron and diabetes risk. Cell Metab. (2013) 17:17–41. 10.1016/j.cmet.2013.02.007 23473030 PMC3648340

[109] Aigner E Felder TK Oberkofler H Hahne P Auer S Soyal S. Glucose acts as a regulator of serum iron by increasing serum hepcidin concentrations. J Nutr Biochem. (2013) 24:24–7. 10.1016/j.jnutbio.2012.02.017 22819549

[110] Vecchi C Montosi G Garuti C Corradini E Sabelli M Canali S. Gluconeogenic signals regulate iron homeostasis via hepcidin in mice. Gastroenterology (2014) 146:146–9. 10.1053/j.gastro.2013.12.016 24361124 PMC3989026

[111] Backe MB Moen IW Ellervik C Hansen JB Mandrup-Poulsen T. Iron regulation of pancreatic beta-cell functions and oxidative stress. Annu Rev Nutr. (2016) 36:36–73. 10.1146/annurev-nutr-071715-050939 27146016

[112] Tuomainen TP Nyyssönen K Salonen R Tervahauta A Korpela H Lakka T. Body iron stores are associated with serum insulin and blood glucose concentrations. Population study in 1,013 eastern finnish men. Diabetes Care (1997) 20:20–8. 9051399 10.2337/diacare.20.3.426

[113] Fernández-Real JM López-Bermejo A Ricart W Fernandez-Real JM Lopez-Bermejo A Ricart W. Cross-talk between iron metabolism and diabetes. Diabetes (2002) 51:51–54. 10.2337/DIABETES.51.8.2348 12145144

[114] De Sanctis V Soliman A Yassin M Iron overload and glucose metabolism in subjects with beta-thalassaemia major: an overview. Curr Diabetes Rev. (2013) 9:9–41. 10.2174/1573399811309040005 23687960

[115] Carlos AR Weis S Soares MP. Cross-regulation of iron and glucose metabolism in response to infection. Biochemistry (2017) 56:56–4. 10.1021/acs.biochem.7b00728 29052977

[116] Sanchez KK Chen GY Schieber AMP Redford SE Shokhirev MN Leblanc M. Cooperative metabolic adaptations in the host can favor asymptomatic infection and select for attenuated virulence in an enteric pathogen. Cell (2018) 175:175–58.e15. 10.1016/j.cell.2018.07.016 30100182 PMC6447043

[117] Lecube A Hernández C Genescà J Simó R Glucose abnormalities in patients with hepatitis C virus infection: epidemiology and pathogenesis. Diabetes Care (2006) 29:29–9. 10.2337/diacare.2951140 16644655

[118] Langley RJ Tsalik EL van Velkinburgh JC Glickman SW Rice BJ Wang C. An integrated clinico-metabolomic model improves prediction of death in sepsis. Sci Transl Med. (2013) 5:5ra95. 10.1126/scitranslmed.3005893 23884467 PMC3924586

[119] Figueiredo RT Fernandez PL Mourao-Sa DS Porto BN Dutra FF Alves LS. Characterization of heme as activator of Toll-like receptor 4. J Biol Chem. (2007) 282:282–9. 10.1074/jbc.M610737200 17502383

[120] Raetzsch CF Brooks NL Alderman JM Moore KS Hosick PA Klebanov S. Lipopolysaccharide inhibition of glucose production through the Toll-like receptor-4, myeloid differentiation factor 88, and nuclear factor kappa b pathway. Hepatology (2009) 50:50–600. 10.1002/hep.22999 19492426 PMC2822400

[121] Yin L Wu N Curtin JC Qatanani M Szwergold NR Reid RA. Rev-erbalpha, a heme sensor that coordinates metabolic and circadian pathways. Science (2007) 318:318–9. 10.1126/science.1150179 18006707

[122] Deutschman CS Andrejko KM Haber BA Bellin L Elenko E Harrison R. Sepsis-induced depression of rat glucose-6-phosphatase gene expression and activity. Am J Physiol. (1997) 273:273–18. 10.1152/ajpregu.1997.273.5.R1709 9374814

[123] Yki-järvinen H Sammalkorpi K Koivisto VA Nikkilä EA. Severity, duration, and mechanisms of insulin resistance during acute infections^*^. J Clin Endocrinol Metab. (1989) 69:69–23. 2666428 10.1210/jcem-69-2-317

